# AI-Assisted Formative Assessment in Clinical Education: From Algorithms to Agency

**DOI:** 10.2196/93710

**Published:** 2026-06-04

**Authors:** Quang Thanh Nguyen, Thuy Minh Ha, Tina Mai

**Affiliations:** 1 College of Health Sciences, VinUniversity, Da Ton, Gia Lam, Hanoi, Hanoi, 100000, Vietnam, 84 89902123; 2 Department of Surgery, Vietnam National Children's Hospital, Hanoi, Hanoi, Vietnam; 3 Royal College of Surgeons in Ireland and University College Dublin Malaysia Campus, George Town, Penang, Malaysia; 4 Center for Medical and Health Sciences Education, University of Auckland, Auckland, New Zealand; 5 School of Medicine, University of California, San Francisco, San Francisco, CA, United States

**Keywords:** artificial intelligence, AI, formative assessment, feedback, medical education, clinical education

## Abstract

Artificial intelligence (AI) is rapidly reshaping clinical education by embedding assessment and feedback into everyday learning activities. Medical students can now use machine learning dashboards, generative AI, large language models, and emerging agentic systems to practice clinical reasoning, communication, and procedural skills while receiving individualized feedback within seconds. However, the availability of more data and more feedback does not necessarily produce better learning. This Viewpoint is intended for clinical educators, assessment leaders, curriculum committees, faculty developers, and institutional leaders who must decide how AI should be used in formative activities without reducing education to automated scoring. AI-assisted formative assessment is defined in this paper as the intentional use of AI tools to generate, organize, and support interpretation of performance information for learning rather than grading. Its distinctive contribution lies in the scale, adaptivity, conversational simulation, pattern detection, and possible autonomy of AI systems. However, AI outputs become formative only when learners and educators interpret them critically, judge their trustworthiness, and translate them into a small number of focused follow-on learning actions. This paper synthesizes the current evidence base while noting that much of it remains early, heterogeneous, and concentrated in short-term or single-setting studies. It examines key risks, including hallucination, automation bias, epistemic overtrust, hidden curricular effects, and broader concerns related to professional identity, power asymmetries, data privacy, and inequitable access. It also presents context-specific implementation examples for preclinical case-based learning, communication and objective structured clinical examination preparation, procedural skill laboratories, clerkship learning, and programmatic assessment portfolios, together with practical implications for faculty development, institutional governance, and phased local implementation. As a Viewpoint rather than an empirical study or systematic review, the framework and examples should be interpreted as evidence-informed design propositions that require local evaluation and validation. Overall, the value of AI-assisted formative assessment depends less on the volume of AI-generated feedback than on educational designs that preserve learner agency, professional judgment, and human accountability.

## Introduction

Formative assessment has always depended on more than the delivery of comments or scores. It requires learners to understand the gap between current and desired performance, receive information that guides improvement, and have opportunities to act on that information [1,2]. In clinical education, these conditions have been difficult to achieve because observation by experienced clinicians is limited, learning encounters are unpredictable, and faculty time is scarce. Artificial intelligence (AI) now changes these conditions by making feedback available during routine learning activities, often at a scale and speed that human educators cannot match.

Recent reviews show rapid expansion of AI applications in medical education, including adaptive learning, automated assessment, simulation, virtual patients, clinical reasoning support, and curriculum reform [3-5]. The scale of this change is significant. A medical student studying late at night can now log into an AI platform, run complex clinical scenarios, and receive individualized feedback that highlights specific areas for improvement within seconds. Unlike a textbook, the system interacts. Unlike a busy attending physician, it has continuous availability. Services that once required coordinated faculty schedules are now available on demand.

These developments extend beyond efficiency. Technology is evolving from static multiple-choice engines into dynamic partners. Machine learning algorithms and large language models (LLMs) now generate high-fidelity virtual patients, analyze procedural performance, and synthesize vast stores of medical knowledge in real time [3,4]. Generative AI has further accelerated these trends by enhancing scalability and enabling more personalized learning experiences [4]. These capabilities create new opportunities for safe, deliberate practice. They also create a pedagogical paradox: learners and faculty may receive more performance data while having less clarity about what to trust, what actions to follow, and who remains accountable for educational decisions. Most existing reports catalog tools and attitudes or describe broad opportunities and risks yet offer limited guidance for clinical teachers on how to turn AI outputs into meaningful assessment for learning [3-5].

Therefore, the gap addressed in this Viewpoint is not whether AI can generate feedback but how this information can be curated into assessment for learning. We define AI-assisted formative assessment, distinguish relevant types of AI tools, summarize the evidence base and its limitations, analyze ethical and practical risks, clarify the role of AI-assisted formative assessment within programmatic assessment, and propose actionable implementation suggestions for specific curriculum contexts. This paper is intended for clinical educators, assessment leaders, curriculum committees, faculty developers, and institutional leaders in health professions education. Its practical concern is how medical schools and clinical training programs should design AI-assisted formative activities so that AI-generated information supports learning, professional judgment, and responsible clinical practice. As a Viewpoint rather than an empirical study or systematic review, this paper offers an evidence-informed conceptual framework and implementation heuristics, not validated protocols or original outcome data.

## What Counts as AI-Assisted Formative Assessment?

The term “AI” is often used broadly in medical education, but this lack of precision can obscure important differences between tools [6]. These distinctions matter because different AI systems create different educational possibilities, risks, and responsibilities [7]. The updated Organisation for Economic Co-operation and Development definition describes an AI system as a machine-based system that can infer from inputs how to generate outputs such as predictions, content, recommendations, or decisions with varying degrees of autonomy and adaptiveness [8]. Within clinical education, AI-assisted formative assessment may involve several types of AI, but these systems should not be treated as interchangeable [9].

Some systems are primarily analytic or predictive, processing learner performance data to generate scores, classifications, patterns, or recommendations. Examples include adaptive learning platforms that identify knowledge gaps, simulation dashboards that quantify procedural efficiency or safety, and automated systems that flag recurrent errors. These precision education tools may uncover nuanced learner patterns and support personalization at scale [9,10]. However, analytic systems generally lack the open-ended interactivity of generative models. Although they may reveal patterns that are difficult for human observers to detect, they usually do not engage learners in open-ended dialogue or independently plan learning activities.

Other systems, particularly generative AI and LLMs, produce text, dialogue, explanations, simulated patient responses, or feedback narratives. Their educational value lies not only in the speed of feedback generation but also in their interactivity. Learners can ask follow-up questions, test alternative reasoning pathways, rehearse clinical conversations, and compare different approaches to a case [4,11]. At the same time, these systems can generate plausible but unsupported explanations. A recent scoping review identified hallucinations and factually incorrect medical content as recurring risks in medical education applications [12]. Therefore, the use of AI systems in formative assessment requires careful verification, interpretation, and evaluative judgment, including the ability to critically assess both the AI output and one’s own clinical reasoning [13,14].

Emerging agentic AI systems represent another category. These systems may pursue goals across multiple steps, select tasks, call external tools, retrieve resources, and personalize learning pathways with greater autonomy. They may eventually support tutoring or coaching functions, although this remains more prospective than established in medical education [15]. However, their use in assessment requires stronger safeguards and clear governance structures because they may influence not only the content of feedback but also what learners practice, what evidence is collected, and which learning priorities are emphasized [6,7].

Building on formative assessment scholarship, AI-assisted formative assessment is conceptualized in this paper as the intentional use of AI tools to generate, organize, and support interpretation of performance information for the purpose of improving learning rather than grading [16,17]. Therefore, it is not synonymous with automated feedback, automated scoring, or AI-generated comments. It becomes formative only when learners and educators use AI-generated information to understand performance and decide what to do next [2,17]. The same AI output could be formative if used in a coaching conversation, summative if used to determine progression, or harmful if treated as an unquestioned verdict [18].

The conceptual contribution of AI-assisted formative assessment is that AI changes the ecology of formative assessment. Traditional formative assessment often depends on episodic human observation and scheduled feedback encounters, whereas AI-assisted formative assessment may enable more continuous, learner-initiated, and responsive cycles of practice and feedback across repeated attempts [9,19]. For example, a student may rehearse a difficult communication scenario in an AI-supported simulation, receive immediate feedback, revise the approach, and repeat the encounter before meeting with a faculty coach. Similarly, a procedural simulator may identify repeated patterns in performance that can inform subsequent deliberate practice. These functions are meaningful only when they support rather than replace learner judgment [13]. Medical students’ experiences of feedback suggest that interpretation and uptake are central to whether feedback becomes educationally useful [20]. Therefore, AI-assisted formative assessment should be understood as a strategy for developing feedback agency, meaning the learner’s capacity to interpret feedback, judge its trustworthiness, and translate it into action, rather than simply as a way of increasing feedback volume [1,21].

This emphasis on interpretation, judgment, and action also helps distinguish AI-assisted formative assessment from adjacent frameworks. AI-assisted formative assessment sits alongside but is not identical to learning analytics, AI-supported feedback, and programmatic assessment. Learning analytics mainly emphasizes detecting and visualizing patterns in learner data [9,10]. AI-supported feedback refers more broadly to AI-generated comments, scores, or suggestions that may or may not be used formatively [4,22]. Programmatic assessment is a broader system in which evidence is collected across multiple occasions, methods, and contexts, with low-stakes information used to support learning and aggregated evidence used for higher-stakes decisions [23]. AI-assisted formative assessment contributes a narrower pedagogic lens by focusing on how AI-generated information is interpreted and translated into focused subsequent actions for learning. Accordingly, AI-generated feedback from a virtual patient, simulation dashboard, or LLM-based reasoning exercise should usually function as one low-stakes source of information within a broader assessment system [7,10,14,23].

## Evidence Base and Current Limitations

The evidence supporting AI-assisted formative assessment is promising but still uneven. Broad reviews document increasing use of AI across medical education, including applications in teaching, assessment, simulation, clinical reasoning, and learning analytics [5,22,24]. These syntheses are valuable for mapping the field, but most are scoping, narrative, or integrative reviews rather than comparative evaluations, so they identify domains of use more readily than they establish educational effectiveness. Reviews and curriculum-focused studies on generative AI similarly describe potential benefits for personalized learning, content generation, simulated dialogue, and feedback while emphasizing challenges related to accuracy, bias, transparency, privacy, academic integrity, and educational governance [4,25].

Several empirical studies illustrate the potential of AI-assisted formative assessment. Stronger empirical evidence currently comes from bounded, single-setting studies, including simulation-based evaluations and at least one randomized controlled trial [26-28]. In surgical simulation, the Virtual Operative Assistant used explainable machine learning to translate raw simulator data into feedback aligned with expert performance benchmarks, supporting more targeted practice in technical skill training [26]. In undergraduate medical education, an LLM-based patient simulation and structured feedback intervention was evaluated in a randomized controlled trial and reported short-term support for clinical decision-making practice among medical students [27]. AI-driven personalized learning platforms have also been studied for their effects on medical students’ learning performance, engagement, and self-directed learning [28].

However, existing literature remains limited and early in development. Many studies are of short-term scope; tool specific; and focused on usability, learner satisfaction, or immediate test outcomes rather than durable changes in clinical reasoning, transfer to workplace performance, patient-centered communication, or professional identity formation. Scoping work on AI-driven virtual patients for communication skill training suggests that these tools may benefit learners, but the evidence of effectiveness remains limited and requires stronger educational design, theory-informed evaluation, and stakeholder involvement [29]. Outcome measures are also heterogeneous, making cross-study comparison difficult. Therefore, claims that AI-assisted formative assessment may support feedback agency, self-regulated learning, clinical reasoning, or broader assessment system change should be interpreted as plausible mechanisms or design hypotheses informed by prior literature rather than outcomes demonstrated in this manuscript. Accordingly, the framework and examples offered below are intended as design propositions for local testing, not as validated protocols or implementation recipes.

## Ethical and Practical Risks Requiring Design Controls

The main risks of AI-assisted formative assessment are not limited to technical inaccuracy. They involve learner trust in feedback, institutional use of learner data, and the potential for AI systems to reshape educational relationships. These risks are particularly important because formative assessment depends on psychological safety, learner agency, and trust between learners and educators.

One concern is epistemic overtrust. AI-generated feedback may appear authoritative because it is fluent, immediate, personalized, or numerically precise. Therefore, learners may accept AI outputs even when the underlying reasoning is incomplete, biased, or unsupported [4,6,30]. This risk is especially relevant for LLM-based feedback, where plausible explanations may not be reliably grounded in clinical evidence. Therefore, AI-assisted formative assessment activities should require learners to verify AI claims against clinical guidelines, local protocols, patient context, and faculty judgment. Verification should not be treated as an optional technical skill but as part of clinical reasoning and professional formation.

A second concern is automation bias and metric gaming. Learners may begin to optimize their performance for what the AI system measures rather than for what clinical competence requires. A procedural simulator may reward speed or economy of movement while insufficiently capturing tissue handling, situational awareness, communication, or teamwork. A virtual patient platform may reward diagnostic completeness while failing to adequately assess empathy, cultural humility, shared decision-making, or nonverbal communication. If educators do not explicitly address these limitations, AI-assisted formative assessment may create a hidden curriculum in which machine-measurable performance is mistaken for professional competence.

A third concern involves professional identity and power. If AI feedback is positioned as more objective than human judgment, learners may experience faculty coaching as secondary or feel that their development is being monitored by systems they cannot question. This is particularly problematic when AI dashboards are visible to supervisors, when learner data are stored without clear boundaries, or when AI-generated evidence is discussed in assessment meetings without opportunities for learner explanation. Research on AI-based clinical decision support suggests that trust and professional identity are shaped by whether users can understand, contest, and contextualize AI outputs [30,31]. In educational settings, these dynamics suggest plausible downstream risks for professional identity, supervisory relationships, and assessment culture even though such effects have not yet been demonstrated directly in AI-assisted formative assessment studies [14,30,31].

A fourth concern is institutional inequity and data governance. Students with access to paid AI tools, stronger digital literacy, better language proficiency, or more supportive faculty may benefit more from AI-assisted formative assessment than their peers. Institutions also need explicit rules for patient data, learner data, vendor access, data retention, algorithmic bias monitoring, and the boundary between low-stakes coaching and high-stakes progression decisions [6,32,33]. Without such structures, AI-assisted formative assessment may widen existing inequities, compromise privacy, or undermine trust in assessment systems.

These risks are not an argument against AI-assisted formative assessment. Rather, they define the minimum safeguards for responsible use: governance, human review, equity, and explicit limits on higher-stakes use [6,7,31-33]. The next section translates these safeguards into practical design and implementation considerations.

## Designing and Implementing AI-Assisted Formative Assessment in Specific Curriculum Contexts

### Implementation Design and Expectations

Implementation of AI-assisted formative assessment should begin with the curriculum problem rather than with the AI tool. In practical terms, educators should first identify the learning outcome, then define the learner task, specify what the AI system will contribute, clarify the human educator’s role, and require a concrete learner action at the end of the activity. This design logic is important because feedback becomes formative only when learners can compare their current performance with expected standards, identify the gap, and use that information to improve subsequent performance [2,34-36]. It is also consistent with the broader argument that AI implementation in health professions education should be guided by educational purpose, human oversight, and governance rather than by technology adoption alone [6,7,25].

### Learner Routines and Action Commitments

Across curriculum contexts, a useful learner routine is to attempt, compare, critique, and act. The learner first completes the task independently so that their own reasoning, performance, or communication strategy is visible before AI feedback is introduced [35,37]. The learner then compares the response with AI-generated feedback and critiques both sources of information, which supports feedback literacy and evaluative judgment [1,13,21]. The final step is to identify one or two specific changes for the next case, encounter, or simulation because feedback has limited educational value unless it closes the loop and changes subsequent learning behavior [14,17,37].

### Preparing Learners for Critical Use of AI

This learner routine requires explicit preparation. Students should be taught how to formulate prompts, judge the plausibility of AI-generated feedback, verify sources, recognize hallucinations, manage uncertainty, and translate feedback into learning plans. This preparation is particularly important for LLMs because they can produce fluent and confident responses even when outputs are incomplete, inaccurate, or unsupported [4,11]. One recent scoping review identified hallucinations and medically incorrect content as important risks in undergraduate medical education applications, underscoring the need for verification routines in educational use [12]. Learners should also be taught boundaries for acceptable AI use, especially that identifiable patient information should not be entered into unapproved systems because privacy, data security, and secondary data use are central governance risks [6,33,38].

### Faculty Calibration and Debriefing

Faculty preparation should be built into the same curriculum design process rather than treated as a separate technical training exercise. Educators need sufficient AI literacy to select tools that fit learning outcomes, recognize limitations in AI outputs, and help learners interpret feedback in relation to clinical reasoning and professional values [9,25,32]. Recent work on AI competencies in medical education emphasizes skills in AI literacy, ethical awareness, data use, critical appraisal, and human oversight [39,40]. Therefore, faculty development should focus on calibration and debriefing: educators can examine an AI-generated critique of a learner’s performance; identify what is accurate, what is missing, and what may be misleading; and then convert the output into coaching questions [6,7,15,41,42]. This preserves the relational function of feedback because effective feedback depends not only on information delivery but also on dialogue, interpretation, and learner uptake [20,21]. It also keeps faculty in an interpretive and coaching role rather than a supervisory role over automated scoring.

### Institutional Implementation Model

Institutional preparation should be embedded before AI-assisted formative assessment is scaled across a curriculum. Institutions should specify which AI tools are approved, what data may be entered, who can access learner outputs, and how long learner data are retained. They should also clarify whether vendors may use submitted data for model training and how AI-generated evidence may be used in assessment conversations [6,7,33]. A pragmatic model is to begin with low-stakes pilots in one or two activities, prespecify local evaluation indicators, review unintended consequences; and scale only after governance, faculty capacity, and equity safeguards are in place [6,7,25,33]. Feasibility will also depend on faculty workload for calibration and debriefing, local technical support, availability of approved tools, and willingness of faculty and learners to adopt new workflows. These constraints may justify slower scale-up or narrower pilots even when the educational rationale is strong. The boundary between formative and summative use should be explicit because programmatic assessment requires careful interpretation of evidence across multiple occasions, methods, and contexts rather than reliance on a single score or data source [16,23]. Learners should be able to question AI-generated feedback and request human review when AI-derived evidence is discussed in assessment meetings because trust in AI-supported systems depends on whether users can understand, contest, and contextualize AI outputs [14,30,31].

### Equity and Scale-Up Safeguards

Equity should be treated as part of implementation design rather than as an afterthought. If AI-assisted formative assessment becomes part of the formal curriculum, institutions should provide access to approved tools instead of leaving students to purchase or select AI systems individually because unequal access to AI tools and digital infrastructure may widen educational disparities [10,32]. Institutions should also monitor whether AI feedback performs differently across learner characteristics such as language background, disability, gender, race, socioeconomic status, prior digital experience, or familiarity with prompt-based tools [7,10]. Bias monitoring and impact assessment are recommended components of responsible AI risk management [33]. Curriculum committees should also examine whether AI-assisted formative assessment activities remain aligned with learning outcomes, whether learners experience them as supportive rather than surveillant, and whether faculty have enough time to debrief outputs meaningfully because AI systems perceived as opaque or controlling may threaten trust and professional identity [10,30,31].

### Focused Action Rather Than Data Accumulation

Across contexts, AI-assisted formative assessment should end with one or two specific changes for the next task or encounter rather than a long list of AI-generated observations [17,34,37]. Table 1 illustrates how this principle can be operationalized across different curriculum settings.

Finally, AI-assisted formative assessment should be evaluated based on educational outcomes rather than technology adoption metrics alone. Counts of prompts submitted, comments generated, or dashboard views do not necessarily reflect learning [9]. Before scaling, local pilots should prespecify a small evaluation set, such as learner participation, quality of action plans, change between repeated attempts, quality of dialogue between faculty and learners, learner trust, faculty time burden, equity of access, and unintended effects on assessment culture. This phased piloting approach is consistent with recent road map work emphasizing trust building, equity, and staged implementation [19]. These indicators align more closely with feedback literacy and self-regulated learning than simple measures of technology use [1].

**Table 1.** Context-specific implementation of artificial intelligence (AI)–assisted formative assessment (AFA).

Curriculum context	Curriculum problem	AFA activity and learner action	Faculty role	Institutional safeguard
Preclinical case-based diagnostic reasoning	Students may receive limited individualized feedback on diagnostic reasoning before clinical rotations.	Learners generate their own differential diagnosis and management plan, then ask an approved LLM^a^ for alternatives. They classify each suggestion as accepted, modified, rejected, or uncertain and justify the decision using guidelines or local protocols.	Debrief how the AI output affected reasoning, uncertainty, and premature closure.	Use approved tools, exclude identifiable patient data, and require source checking against trusted clinical guidance.
Communication skills and OSCE^b^ preparation	Students need repeated low-stakes practice in history taking, explanation, empathy, and shared decision-making.	Learners practice with an AI virtual patient, review the transcript, and identify one communication strength and one behavior to improve.	Use selected transcripts or reflections to coach empathy, uncertainty, cultural sensitivity, and patient-centered language.	Do not let AI ratings replace faculty, standardized patient, or patient ratings, especially for nonverbal behavior, professionalism, and relational care.
Procedural or surgical skill laboratory	Students need repeated practice and timely feedback on technical performance without compromising patient safety.	Simulation software or AI analytics identify efficiency, instrument path, safety, or error patterns. Learners select one metric for deliberate practice and repeat the task.	Confirm that the metric is clinically meaningful and prevent speed from being rewarded over safety.	Validate metrics locally against expert performance and patient safety priorities before using them in assessment conversations.
Clerkship and workplace-based learning	Workplace feedback may be fragmented, delayed, or difficult for learners to synthesize across supervisors and encounters.	Learners use an approved AI tool to organize reflections, compare management plans with local guidelines, or summarize recurring workplace-based assessment themes. They bring one improvement goal to coaching.	Review the learner’s interpretation of AI summaries rather than forwarding unexamined outputs to committees.	Prohibit identifiable patient data in public tools and define access, consent, and storage rules for AI-generated summaries.
Programmatic assessment portfolio	Learners and coaches may struggle to identify developmental patterns across multiple low-stakes data points.	AI helps visualize patterns across practice attempts, reflections, OSCEs, and workplace-based assessment comments. Learners use these patterns to prepare a short improvement plan.	Triangulate AI-generated patterns with workplace observations, OSCE performance, portfolio evidence, and learner explanation.	Require transparent criteria, human review, and an appeal process for any higher-stakes use.

a LLM: large language model.

b OSCE: objective structured clinical examination.

## Limitations

This Viewpoint is conceptual and synthetic rather than empirical. It does not report original data, follow a systematic review methodology, or test the proposed framework or implementation examples. The framework and Table 1 are intended as design heuristics to support local planning, not as validated protocols. Therefore, claims about possible effects on feedback agency, self-regulated learning, clinical reasoning, or workplace performance should be interpreted as evidence-informed possibilities rather than demonstrated outcomes in this paper. In addition, institutional feasibility will vary according to local resources, learner level, assessment culture, faculty capacity, governance, and regulatory context. For that reason, local pilots should include predefined outcomes, equity monitoring, and mechanisms for human review before wider scale-up.

## Conclusions

AI-assisted formative assessment offers a meaningful opportunity to strengthen assessment for learning in clinical education, but its value depends on careful human oversight. AI can deliver immediate, scalable, and personalized feedback, yet these tools should complement rather than replace educators’ professional judgment. Therefore, the framework proposed in this paper should be read as an evidence-informed guide for design and local testing rather than as proof of educational effectiveness. The future of AI-assisted formative assessment should be judged not by the sophistication of algorithms or the volume of data produced but by whether these systems help learners understand their decisions; refine their reasoning; and act on focused feedback in ways that preserve agency, professional values, and human accountability.

## Abbreviations

AI: artificial intelligence
LLM: large language model
